# Impact of *Ninjin'Yoeito* on Fatigue in Patients Receiving Nab-Paclitaxel Plus Gemcitabine Therapy: A Prospective, Single-Arm, Phase II Open Label, Nonrandomized, Historically-Controlled Study

**DOI:** 10.1016/j.curtheres.2020.100605

**Published:** 2020-09-15

**Authors:** Ken-ichi Okada, Manabu Kawai, Seiko Hirono, Motoki Miyazawa, Yuji Kitahata, Ryohei Kobayashi, Masaki Ueno, Shinya Hayami, Toshio Shimokawa, Hiroki Yamaue

**Affiliations:** a Second Department of Surgery*,* Wakayama Medical University*,* Wakayama*,* Japan; b *Clinical Study Support Center,* Wakayama Medical University*,* Wakayama*,* Japan

**Keywords:** Fatigue, Japanese herbal medicine, Nab-paclitaxel plus gemcitabine, *Ninjin'yoeito*, Supportive therapy

## Abstract

**Background:**

*Ninjin'yoeito*, a traditional Japanese herbal medicine, is used to prevent fatigue, loss of appetite, and coldness of limbs. Fatigue is an especially common issue during chemotherapy and can affect quality of life and the ability to complete scheduled treatment.

**Objectives:**

This prospective exploratory trial evaluates the efficacy of *ninjin'yoeito* for fatigue in patients undergoing nab-paclitaxel plus gemcitabine therapy for unresectable pancreatic cancer. The primary end point was evaluation of fatigue according to Functional Assessment of Chronic Illness Therapy-Fatigue score during 2 courses of nab-paclitaxel plus gemcitabine therapy. Secondary end points included evaluation of dose intensity, appetite loss using numerical rating scale, and peripheral neuropathy using a patient neurotoxicity questionnaire.

**Methods:**

We compared data from this interventional trial with a prior observational trial without administration of *ninjin'yoeito* with identical definition of end points (UMIN000021758). Thirty patients were required by the study.

**Results:**

Threshold mean of Functional Assessment of Chronic Illness Therapy-Fatigue score across 8 weeks during chemotherapy was under 5.3 (*P* = 0.002). Secondary end points did not reveal any specific patterns in appetite loss or degree of pain. No significant changes in patient neurotoxicity questionnaire concerning sensory/motor disorders were observed, but the mean (SD) incidence of patients with sensory disturbance was higher between the fifth and eighth weeks (8.8 [1.26]) than during the first and fourth weeks (4.8 [0.96]) (*P* = 0.003). Clinically significant adverse reactions of *ninjin'yoeito* were not observed.

**Conclusions:**

*Ninjin'yoeito* may be useful for improving the symptoms of fatigue caused by nab-paclitaxel plus gemcitabine in patients with unresectable pancreatic cancer. UMIN Clinical Trials Registry identifier: UMIN000025606. (*Curr Ther Res Clin Exp*. 2020; 81:XXX–XXX)

## Introduction

Pancreatic cancer has an extremely poor prognosis. By 2030 it is expected to surpass breast, prostate, and colorectal cancers to become the second most common cause of cancer-related death.^1^ Although approximately 20% of patients have borderline resectable or resectable pancreatic cancer at diagnosis, cure is exceedingly rare, even after curative-intent surgery; the 10-year overall survival is only 4%.^2^ Most patients with advanced pancreatic carcinoma report fatigue. Frequent use of multiple chemo-agent regimen correlates with higher levels of reported fatigue.^3^, ^4^, ^5^ Incapacitating fatigue often leads to poor quality of life (QOL) in patients with pancreatic cancer and it can shorten the duration of treatment because of poor tolerance to standard therapy. To improve the QOL and sustainability of patients who receive these chemotherapies, supporting therapy based on evidence is urgently needed. Nab-paclitaxel plus gemcitabine is a first-line therapy and a promising treatment for unresectable pancreatic cancer (URPC), including metastatic/locally advanced pancreatic cancer.^3^^,^^4^ In an international multicenter randomized Phase III study, frequency rates of treatment-related adverse events grade ≥3 were reported in response to nab-paclitaxel plus gemcitabine therapy, including neutropenia (38%), leucopenia (31%), fatigue (17%), and peripheral neuropathy (17%).^3^ The frequency and degree of adverse events during chemotherapy affect QOL of patients with URPC and thus the sustainability of therapy. Fatigue, for example, impairs patients’ energy during chemotherapy, a challenge to the sustainability of therapy.

Natural products can play a critical role in the treatment of various types of cancers.^6^, ^7^, ^8^ *Ninjin'yoeito* (NYT), for example, is a traditional Japanese herbal medicine and is used to prevent fatigue, loss of appetite, and coldness of limbs, among other uses.^9^^,^^10^ NYT extract granules (*Ninjin'yoeito* TJ-108; Tsumura & Co, Tokyo, Japan) are manufactured as dried extract of the following mixed crude drugs: 12.9% rehmannia root, 12.9% Japanese angelica root, 12.9% Atractylodes rhizome, 12.9% poria sclerotium, 9.7% ginseng, 8.1% cinnamon bark, 6.5% polygala root, 6.5% peony root, 6.5% *Citrus unshiu* peel, 4.8% astragalus root, 3.2% *Glycyrrhiza*, and 3.2% *Schisandra* fruit.^11^ NYT includes constituents that relieve the fatigue caused by chemotherapy.^12^, ^13^, ^14^, ^15^ Among patients who receive nab-paclitaxel plus gemcitabine therapy, QOL is often impaired by fatigue and peripheral neuropathy. Balancing subjective and objective assessment of these symptoms is difficult in clinical settings. We previously prospectively assessed the feasibility and validity of patient-based scales in patients with URPC who underwent nab-paclitaxel plus gemcitabine therapy^16^ using Functional Assessment of Chronic Illness Therapy-Fatigue (FACIT-F) questionnaire,^17^ and the Patient Neurotoxicity Questionnaire (PNQ).^18^ The threshold mean of FACIT-F score during chemotherapy was 5.3. We hypothesized that administration of NYT might improve FACIT-F score during chemotherapy.

The current single center, prospective exploratory trial uses FACIT-F scores to evaluate the usefulness of NYT for fatigue in patients undergoing nab-paclitaxel plus gemcitabine therapy for URPC.

## Patients and methods

### Protocol digest

This study was approved by the Wakayama Medical University Hospital Institutional Review Board (No. 1895). We designed 2 studies to evaluate the effect of NYT, an observational study without administration of NYT,^16^ then the current interventional study, in which NYT was administered. No patients were enrolled in both studies. To facilitate comparison of data with this interventional trial registered on the UMIN Clinical Trials Registry (identifier: UMIN000025606), patients with severe comorbidities were excluded, such as severe cardiac/renal failure or bowel obstruction, and patients unable to intake oral medicine. End points in the current study are identical to those in the first observational study so that fatigue can be evaluated in comparison with patients who underwent nab-paclitaxel plus gemcitabine therapy without receiving NYT (identifier: UMIN000021758).

### Ethical approval and consent to participate

All procedures performed in studies involving human participants were in accordance with the ethical standards of the institutional and national research committees and with the 1964 Helsinki declaration and its later amendments or comparable ethical standards. Informed consent was obtained from all individual participants included in the study.

### Product quality and safety management

The current quality standards for traditional Japanese herbal medicines are set based on notification from the Ministry of Health and Welfare in 1980 (Pharmaceutical Trial No. 804) and 1985 (Pharmaceutical Trial No. 2120). Based on this setting, many test items are inspected, such as component quantitative tests by instrumental analysis. Furthermore, as an in-house standard, quality tests related to safety, such as to detect pesticide residues and microorganisms, are also conducted at the Analysis and Formulation Research Center at Tsumura & Co.

### Primary and secondary end points

The primary end point of this study is assessment of fatigue evaluation according to the FACIT-F questionnaire (version 4) and additional concerns (Japanese version) during 2 courses of nab-paclitaxel plus gemcitabine therapy for patients with URPC. Secondary end points include appetite loss, pain, and sensory disorder evaluated by a numerical rating scale (NRS), cumulative sensory/motor neurotoxicity according to PNQ, degree of pain, and sensory disorder according to NRS, toxicity, and adverse effects of chemotherapy using Common Terminology Criteria for Adverse Events (CTCAE), and completion of scheduled chemotherapy.

### Treatment

One cycle of regimen comprised the following: on days 1, 8, and 15 over a 4-week period, enrolled patients were administered a 30-minute intravenous infusion of nab-paclitaxel at a dose of 125 mg/m^2^. This was followed by a 30-minute intravenous infusion of gemcitabine at a dose of 1000 mg/m^2^.^5^ There was 1 week of rest between each cycle. Criteria for restart, dose reduction, and discontinuation of chemotherapy were also as previously reported.^5^ Treatment was repeated until disease progression, if toxicity levels became unacceptable, or when discontinuation was decided either by patient refusal or by the investigators. Where there was no disease progression, patients continued chemotherapy.

### Assessments

Before administration of nab-paclitaxel plus gemcitabine, enrolled patients completed FACIT-F (version 4) and questionnaires on any additional concerns, an NRS test on appetite loss, degree of pain, sensory disorder (cold or burning), and PNQ. The first 2 cycles of continuous regimen comprised an 8-week period of therapy, and questionnaires and tests were completed on registration day and weekly thereafter on days 1, 8, 15, 22, 29, 36, 43, and 50. FACIT-F was assessed by degree, which was converted to numerical values as follows: 0 = not at all, 1 = a little bit, 2 = somewhat, 3 = quite a bit, and 4 = very much. Total values were recorded weekly for each questionnaire. Appetite loss, degree of pain, and sensory disorder were assessed by NRS converted to 0 to 10, cumulative sensory/motor neurotoxicity were assessed with PNQ converted to 0 to 4. Toxicity and adverse effects of chemotherapy were assessed in accordance with CTCAE version 4.0. Complete blood counts and differential count of leukocytes, blood chemical tests, and physical examinations were carried out at least every week until the end of the 2 cycles, then every 2 weeks thereafter. In cases of grade 4 hematological toxicity, re-examination within 4 days was required.

### Eligibility criteria

#### Inclusion criteria

Inclusion criteria were:

• Nab-paclitaxel plus gemcitabine therapy as a first line chemotherapy;
• Eastern Cooperative Oncology Group (ECOG) performance status (PS) of 0 or 1;
• Age ≥20 years;
• Laboratory tests within 14 days of registration: white blood cell count ≥3500/mm^3^ and ≤12,000/mm^3^, neutrophil count ≥1500 /mm^3^, hemoglobin ≥9.0 g/dL, platelet count ≥100,000/mm^3^, total bilirubin ≤2.0 mg/dL (≤3.0 mg/dL in biliary drainage cases), serum creatinine ≤1.2 mg/dL, aspartate aminotransferase, alanine aminotransferase ≤100 IU/L. Patients with URPC during the study period receive nab-paclitaxel plus gemcitabine therapy as a first line chemotherapy in our institute;
• Unresectable pancreatic cancer as defined by National Comprehensive Cancer Network version 2.2016 criteria^19^; and
• Written informed consent to inclusion in this study.

#### Exclusion criteria

Exclusion criteria were:

• Severe comorbidity, such as heart failure, renal failure, or bowel obstruction;
• Pregnancy or expecting pregnancy;
• Active cancer of other organs;
• Intolerance of oral medications;
• Use of Japanese herbal medicines (*kampo*) within a week before registration; and
• Unsuitability due to uncontrollable psychiatric illness.

#### Registration

Eligibility report forms were sent for registration at Wakayama Medical University Clinical Study Support Center. Information regarding the necessary follow-up tests was sent from there to the Second Department of Surgery.

### Treatment method

NYT extract granules for ethical use (Tsumura) were administered orally at a dose of 3 g before meals 3 times daily (total 9 g/day) for 8 weeks from commencement of chemotherapy.

### Discontinuation of protocol treatment criteria

If patients presented grade 3 NYT-related adverse effects (CTCAE version 4.0 criteria) protocol treatment was to be immediately discontinued.

### Data collection

Data were collected prospectively for all patients, including history, physical data, laboratory data, pathologic examination, and patient-based scale parameters.

### Sample size calculation

We conducted an observational study to evaluate fatigue and malaise using FACIT-F in patients with URPC after nab-paclitaxel plus gemcitabine therapy.^16^ Mean (SD) increase in FACIT-F at 8 weeks after chemotherapy was 5.3 (7.2). In the current trial, we expected the mean of this score to be less than half of that of the previous study. We set expected mean (SD) at 2.3 (7.2). The null hypothesis: Mean increase of FACIT-F score within 8 weeks during chemotherapy was 5.3, and alternative hypothesis: Mean of FACIT-F score within 8 weeks during chemotherapy was under 5.3, were assessed using a 1-sample *t* test with significance level of 0.05 and a power of 0.80. The necessary sample size was therefore 26 patients. Total sample size was set at 30 patients to account for deviation.

### Statistical analysis

We evaluated the mean FACIT-F score over 8 weeks of chemotherapy using one sample *t* test with threshold mean of 5.3. These scores were summarized by mean and 95% CI at each time point. In other secondary end points, categorical outcomes were summarized using frequency and percentage and 95% CI was calculated using Clopper-Pearson exact method. Continuous outcomes were median and range. All analyses were performed using the JMP Statistical Software, version 13 (SAS Institute Inc, Cary, NC).

## Results

### Patient characteristics

**Figure 1:**
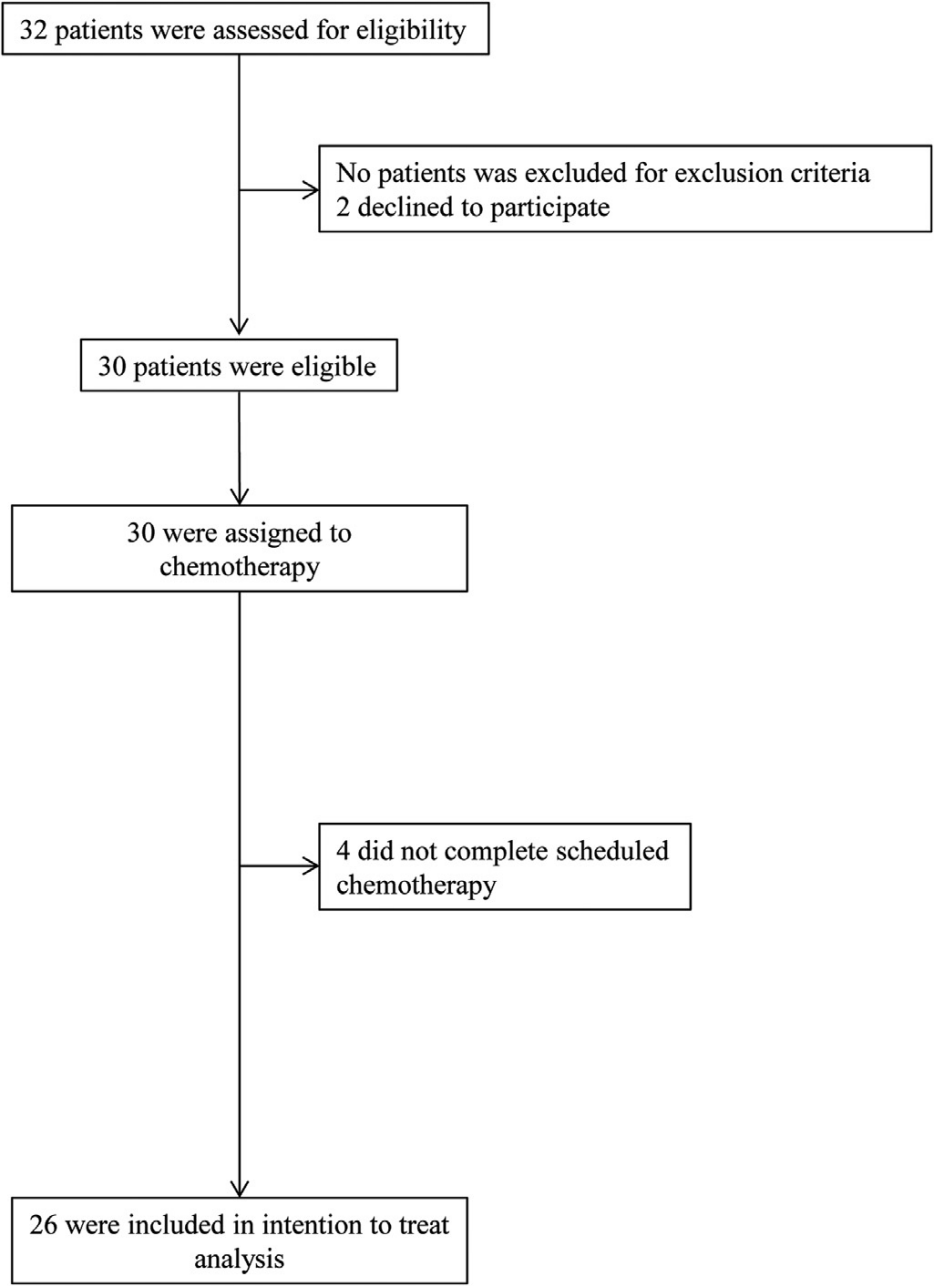
Consolidated Standards of Reporting Trials diagram.

Between March 2017 and August 2018, 32 patients were assessed for eligibility in our hospital. Thirty of these patients were enrolled in the study after 2 patients declined to participate. Four patients (13%) later discontinued the protocol therapy due to disease progression. All 26 patients (87%) completed the protocol therapy and were included in the safety analysis (Figure 1). Patient characteristics at baseline are shown in Table 1. The median age was 73 years (range = 59–79 years), 24 patients had an ECOG PS 0, and 6 patients had an ECOG PS 1. The primary sites of the tumors were the head (57%), body (33%), and tail (10%) of the pancreas. The relative dose intensities of nab-paclitaxel and gemcitabine were 86.9% and 86.5%, respectively.

**Table 1:** Characteristics of patients in a study to evaluate the efficacy of *ninjin'yoeito* for fatigue in patients undergoing nab-paclitaxel plus gemcitabine therapy for unresectable pancreatic cancer.

Characteristic	Result
Baseline	
Male/female sex	17/13*
Age, y	72.5 (59-79)†
Body height, cm	159.0 (139.9-186.0)†
Body weight, kg	56.6 (33.6-92.2)†
Location of pancreatic cancer‡	13/17*
Comorbidity	
Diabetes mellitus	10 (33)§
Hypertension	18 (60)§
PS 0/1	24/6*
Frequency of administration	5.2 (3-6)†
Dose reduction	12 (40)§
Metastatic/locally advanced	23/7*
UICC stage	
III	7 (23)§
IV	23 (77)§
Completion of chemotherapy	26 (87)§

PS = performance status; UICC = Union for International Cancer Control.

⁎ Values are presented as n/n.

† Values are median (range).

‡ Body-tail/head.

§ Values are presented as n (%).

### Study outcomes

#### End points

**Figure 2:**
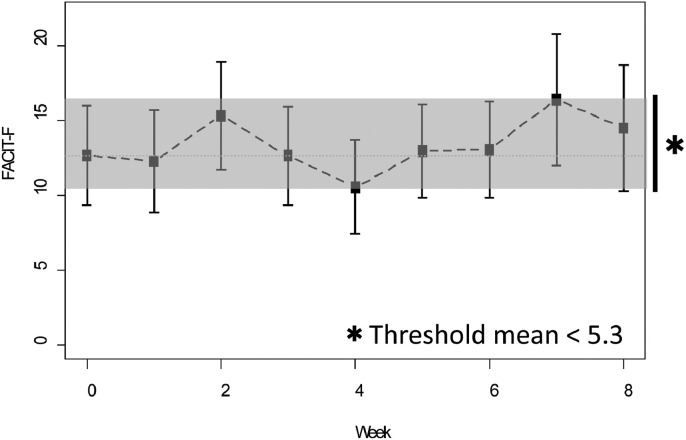
Change in Functional Assessment of Chronic Illness Therapy-Fatigue (FACIT-F) score. FACIT-F was evaluated by the mean of the degrees; each degree was converted to numerical values as follows: 0 = not at all, 1 = a little bit, 2 = somewhat, 3 = quite a bit, and 4 = very much. The *y*-axis represents the total number of values. Total values were recorded weekly for each questionnaire. Threshold mean of FACIT-F score until 8 weeks during chemotherapy was under 5.3 (*P* = 0.002).

Baseline compliance with completion of FACIT-F, NRS, and PNQ revealed favorable data deficit in intention to treat analysis (n = 26): 87% of all patients completed all questionnaires. Table 2 shows patient-reported outcomes by means of FACIT-F fatigue evaluation in this study. Threshold mean of FACIT-F score within 8 weeks during chemotherapy was under 5.3 (*P* = 0.002) (Figure 2).

**Table 2:** Assessment of effectiveness on version 4 of the Functional Assessment of Chronic Illness Therapy-Fatigue (FACIT-F). There were 4 missing cases.

	FACIT-F*		Average increase† (95% CI)	*P* value against threshold (5.3)
	0 wk	8 wk		
Present study	12.6	14.5	1.0	0.002
	(9.3 to 16.0)	(10.3 to 18.7)	(–2.5 to 4.6)	
Previous study	15.2	20.9	5.3	
	(10.4 to 20.0)	(14.1 to 27.7)	(–0.1 to 10.7)	

The threshold mean of FACIT-F score during chemotherapy was 5.3 in the previous study. In the present study, it was decreased significantly.

⁎ Values are presented as mean value (95% CI).

† Values are presented as average increase (95% CI).

**Figure 3:**
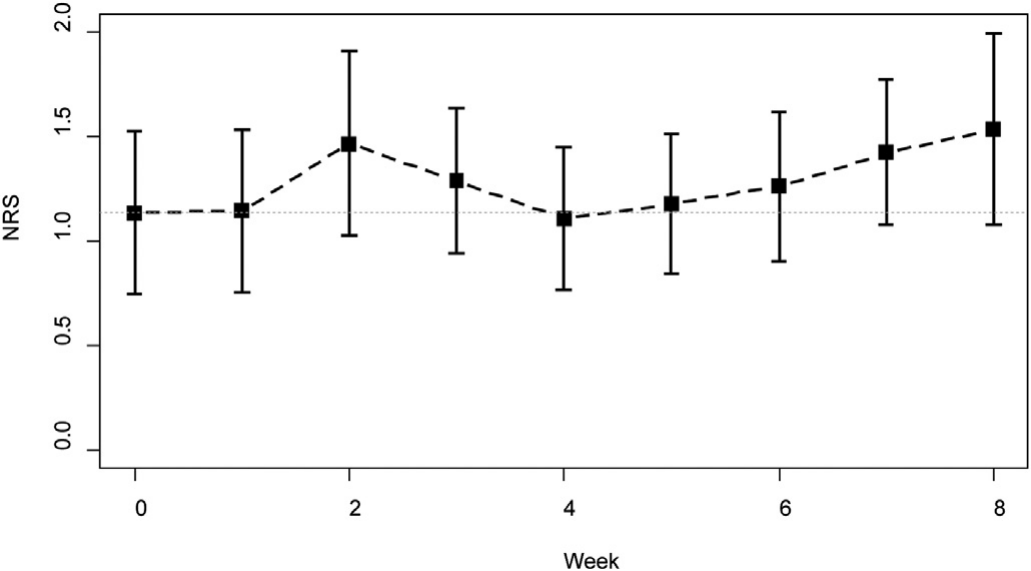
Change in numerical rating scale (NRS) score. Graphical data of appetite loss evaluated by NRS. There were no specific patterns.

Secondary end points did not reveal any specific patterns in appetite loss (Figure 3), or in degree of pain. There was a rapid increase in sensory disorder score during chemotherapy followed by decrease according to evaluation of the number of patients by NRS (not shown). These corresponded with the rest periods. No significant changes in PNQ concerning sensory/motor disorders were observed, but there were more (mean [SD] patients with sensory disturbance between the fifth and eighth weeks (8.8 [1.26]) compared with between the first and fourth weeks (4.8 [0.96]) (*P* = 0.003) (Table 3). Two patients reported burning pain, and incidences of cold sensory disorders increased with the number of administrations.

**Table 3:** Incidence of sensory disorder in patients with unresectable pancreatic cancer (n = 30).

Disorder*	Week								
	0	1	2	3	4	5	6	7	8
No change	21	23	23	19	19	19	16	13	17
Cold sensory disturbance	0	3	2	5	4	6	8	8	8
Burning pain	0	1	2	1	1	1	1	1	2
Deficit	9	3	3	5	6	4	5	8	3
Cases sensory disturbance	0	4	4	6	5	7	9	9	10
Incidence of sensory disturbance (%)†	0	15	15	24	21	27	36	41	37

⁎ Values are presented as number of cases.

† The mean (SD) incidence of patients with sensory disturbance was elevated in fifth to eighth week (8.8 [1.26]) compared with the first to fourth week (4.8 [0.96]) (*P* = 0.003).

#### Adverse events

Table 4 shows the overall rate of grade 3 and 4 toxicity during the protocol treatment (CTCAE version 4.0 criteria). The incidence of fatigue identified with CTCAE grading was low (3.3%) in this study. No grade 4 adverse events were observed in 6 patients (20%) that had neutropenia. One patient had serious adverse effects from febrile neutropenia, otherwise no patients had sepsis, liver abscess, or severe diarrhea grade >3. Patients with grade 3 or 4 neutropenia tended to have a decreasing number of neutrophils toward the end of the second week of each cycle. Clinically significant adverse reactions of NYT such as pseudoaldosteronism, myopathy, hepatic dysfunction, or jaundice were not observed.

**Table 4:** Toxicity following treatment with neoadjuvant nab-paclitaxel plus gemcitabine therapy in patients with unresectable pancreatic cancer (n = 30).

Treatment toxicity*	All grades	Grade 3	Grade 4

Leukopenia	6 (20)	5 (16.7)	0
Anemia	3 (10)	0	0
Thrombocytopenia	10 (33.3)	3 (10)	0
Neutropenia	17 (56.7)	11 (36.7)	6 (20)
Liver dysfunction	1 (3.3)	0	0
Appetite loss	2 (6.7)	0	0
Nausea	1 (3.3)	0	0
Urticaria	5 (16.7)	0	0
Diarrhea	2 (6.7)	0	0
Fatigue	1 (3.3)	0	0
Hyponatremia	1 (3.3)	1 (3.3)	0
Febrile neutropenia	1 (3.3)	0	1 (3.3)
Cholangitis	1 (3.3)	0	0
Interstitial pneumonia	0	0	0
Peripheral sensory neuropathy	1 (3.3)	0	0

⁎ Safety was evaluated in accordance with the Common Terminology Criteria for Adverse Events version 4.0. Values are presented as number of events (%).

## Discussion

Using the key words *ninjinyoeito* or *ninjin'yoeito*, we conducted a database search of PubMed, Cochrane Library, and Evidence Reports of *kampo* Treatment for articles written in English, and Ichushi, J-Stage, and Evidence Reports of *kampo* Treatment for those written in Japanese. The search was restricted to articles published before July 18, 2020. We also performed an additional hand search for recently published articles. To date, no prospective studies have investigated the effect of NYT on fatigue suppression during chemotherapy for pancreatic cancer. The present study suggests that NYT may be useful for improving fatigue symptoms in patients with URPC caused by nab-paclitaxel plus gemcitabine therapy. We prospectively combined subjective and objective assessments of interventional therapy with a traditional Japanese herbal medicine, NYT*,* using FACIT-F questionnaire as a patient-reported outcome measurement. In our clinical experience, we encounter fatigue in patients who undergo chemotherapy for pancreatic cancer more frequently during the first or second course than during the third course and after. Results of weekly assessment of fatigue by FACIT-F during the first 2 cycles of consecutive chemotherapy with NYT was compared with results from an identically designed study without NYT.^16^ The primary end point of evaluation of fatigue according to FACIT-F was met in the present study. We previously demonstrated increase in the mean maximum degree of fatigue from mean baseline values by nab-paclitaxel plus gemcitabine therapy across all categories of the questionnaire.^16^ In the present study, the threshold mean of FACIT-F score within 8 weeks of chemotherapy was significantly lower than that of the previous study.

The most common adverse events associated with chemotherapy agents against pancreatic cancer include hematologic events and gastrointestinal disorders. General disorders, such as fatigue, can also be significant adverse events that impair patients’ ability to continue during chemotherapy and prevent maintenance of dose intensity of chemotherapy. Recent studies have reported favorable survival outcomes by stronger chemotherapy not only for patients with URPC, but also those with borderline resectable pancreatic cancer.^2^ Avoiding dose reduction or discontinuation of treatment due to fatigue is therefore important for extending patient survival, for improving QOL, and for neoadjuvant chemotherapeutic effect. In multiagent regimens for pancreatic cancer, overlooking the incidence of fatigue of patients with URPC is somewhat inevitable.^3^^,^^4^ Cruz et al^20^ investigated potential biomarkers of fatigue, concluding that fatigue induced by chemotherapy in patients with breast cancer is associated with changes in interleukin 1-ra plasma levels and in transforming expression of growth factor-β lymphocyte. However, there are no effective means of treating these general subjective symptoms except for dose reduction or treatment discontinuation. Ito et al^12^ reported that NYT may also be useful for improving the symptoms of subjective fatigue caused by lenalidomide in patients with multiple myeloma.

A possible mechanism of action of NYT in relieving the fatigue caused by chemotherapy could be an effect of 1 of the constituent parts*, Schisandra* fruit *(Schisandra chinensis). Schisandra* fruit has been reported to improve endurance and energy metabolism in exercised rats, and has the potential to upregulate peroxisome proliferator-activated receptor-gamma coactivator-1α in skeletal muscle, which is a key regulator of energy metabolism.^13^ A metabolic mechanism of a polysaccharide from *Schisandra* to relieve chronic fatigue syndrome has been reported.^14^ Alternatively, α-cubebenoate may act as an antifatigue constituent of *Schisandra* through anti-inflammation, and could be of therapeutic use as a treatment for inflammatory diseases such as cancer.^15^

Further studies are needed to clarify the detailed mechanisms behind the antifatigue properties of NYT. Increased treatment duration and sustainability of stronger chemotherapy regimen with NYT may have the potential to lead to favorable survival outcomes.

This study has several limitations. First, neither the people who evaluated the patients and gathered data for the subjective scoring systems used nor the patients who provided the responses were masked as to what the patients had been taking. This is a major study limitation, and it made it highly likely that evaluator and subject biases affected the responses. Similar potential bias may also be a consideration in the assessment of adverse effects. A randomized, double-blind placebo-controlled Phase III trial is therefore needed to validate the evidence of the present study. Other limitations of this study include the small sample size, a single institution-based study, the short observation period, lack of evidence about the subjective QOL improvement in chemotherapy on prognostic survival, lack of subjective data from patients who discontinued the protocol therapy, unknown suitability of timing of intervention, lack of objective discrimination between chemotherapy-related fatigue and cancer-related fatigue by an experienced psychiatrist, and the grading setting of assessment tools.

## Conclusions

NYT may be useful for improving the symptoms of subjective fatigue caused by nab-paclitaxel plus gemcitabine in patients with URPC. The low incidence of fatigue may affect the sustainability of therapy. Additional research in a double-blinded randomized controlled study examining the effects of NYT on fatigue is warranted.

## Appendix Supplementary materials

Image, application 1
