# Supplementary material for: Impact of Ninjin'Yoeito on Fatigue in Patients Receiving Nab-Paclitaxel Plus Gemcitabine Therapy: A Prospective, Single-Arm, Phase II Open Label, Nonrandomized, Historically-Controlled Study
Source: Curr Ther Res Clin Exp. 2020 Sep 15;93:100605. doi: 10.1016/j.curtheres.2020.100605 (PMC7522496; doi:10.1016/j.curtheres.2020.100605)
Supplement: Supplementary file 1 [file mmc1.pdf]

Revised: March 2013 (6th version)

Standard Commodity Classification No. of Japan
875200

- Kampo-preparation-

TSUMURA Ninjin’yoeito Extract Granules for Ethical Use

<ninjin’yoeito>

Storage
Store in light-resistant, air-tight containers.

Expiration date
Use before the expiration date indicated on the container and the outer package.

Approval No.	(61AM)3305
Date of listing in the NHI reimbursement price	October 1986
Date of initial marketing in Japan	October 1986

DESCRIPTION

Composition	9.0 g of TSUMURA Ninjin’yoeito extract granules contains 6.0 g of a dried extract of the following mixed crude drugs.<br>JP Rehmannia Root .... 4.0 g<br>JP Japanese Angelica Root .... 4.0 g<br>JP Atractylodes Rhizome .... 4.0 g<br>JP Poria Sclerotium .... 4.0 g<br>JP Ginseng .... 3.0 g<br>JP Cinnamon Bark .... 2.5 g<br>JP Polygala Root .... 2.0 g<br>JP Peony Root .... 2.0 g<br>JP Citrus Unshiu Peel .... 2.0 g<br>JP Astragalus Root............................. 1.5 g<br>JP Glycyrrhiza ……………………… 1.0 g<br>JP Schisandra Fruit …………………. 1.0 g<br>(JP: The Japanese Pharmacopoeia)	
	Inactive ingredients	JP Magnesium Stearate<br>JP Lactose Hydrate
Description	Dosage form	Granules
	Color	Grayish-brown
	Smell	Characteristic smell
	Taste	Astringent and sweet
	ID code	TSUMURA/108

INDICATIONS

TSUMURA Ninjin’yoeito Extract Granules (hereafter TJ-108) is indicated for the relief of the following symptoms:
Declined constitution after recovery from disease, fatigue and malaise, anorexia, perspiration during sleep, cold limbs, and anemia.

DOSAGE AND ADMINISTRATION

The usual adult dose is 9.0 g/day orally in 2 or 3 divided doses before or between meals. The dosage may be adjusted according to the patient's age and body weight, and symptoms.

PRECAUTIONS

1. **Careful administration (TJ-108 should be administered with care in the following patients.)**
   (1) Patients with an extremely weak gastrointestinal tract [Anorexia, epigastric distress, nausea, vomiting, abdominal pain, diarrhea, etc. may occur.]
   (2) Patients with anorexia, nausea or vomiting [These symptoms may be aggravated.]

2. **Important Precautions**
   (1) When TJ-108 is used, the patient’s “SHO” (constitution/symptoms) should be taken into account. The patient’s progress should be carefully monitored, and if no improvement in symptoms/findings is observed, continuous treatment should be avoided.
   (2) Since TJ-108 contains Glycyrrhiza, careful attention should be paid to the serum potassium level, blood pressure, etc., and if any abnormality is observed, administration should be discontinued.
   (3) When TJ-108 is coadministered with other Kampo-preparations (Japanese traditional herbal medicines), etc., attention should be paid to the duplication of the contained crude drugs.

   SHO: The term “SHO” refers to a particular pathological status of a patient evaluated by the Kampo diagnosis, and is patterned according to the patient’s constitution, symptoms, etc. Kampo-preparations (Japanese traditional herbal medicines) should be used after confirmation that it is suitable for the identified “SHO” of the patient.

3. Drug Interactions

Precautions for coadministration (TJ-108 should be administered with care when coadministered with the following drugs.)

Drugs	Signs, Symptoms, and Treatment	Mechanism and Risk Factors
(1) Preparations containing Glycyrrhiza<br>(2) Preparations containing glycyrrhizinic acid or glycyrrhizinates	Pseudoaldosteronism is likely to occur. Besides, myopathy is likely to occur as a result of hypokalemia.<br>(Refer to the section "Clinically significant adverse reactions".)	Since glycyrrhizinic acid has an accelerating action on the potassium excretion at the renal tubules, an acceleration of decrease in the serum potassium level has been suggested.

4. Adverse Reactions

TJ-108 has not been investigated (drug use investigations, etc.) to determine the incidence of adverse reactions. Therefore, the incidence of adverse reactions is not known.

(1) Clinically significant adverse reactions

1) **Pseudoaldosteronism**: Pseudoaldosteronism such as hypokalemia, increased blood pressure, retention of sodium/body fluid, edema, increased body weight, etc. may occur. The patient should be carefully monitored (measurement of serum potassium level, etc.), and if any abnormality is observed, administration should be discontinued and appropriate measures such as administration of potassium preparations should be taken.

2) **Myopathy**: Myopathy may occur as a result of hypokalemia. The patient should be carefully monitored, and if any abnormality such as weakness, convulsion/paralysis of limbs, etc. are observed, administration should be discontinued and appropriate measures such as administration of potassium preparations should be taken.

3) **Hepatic dysfunction and jaundice**: Hepatic dysfunction and/or jaundice with elevation of AST (GOT), ALT (GPT), Al-P and γ-GTP or other symptoms may occur. The patient should be carefully monitored for abnormal findings. Administration should be discontinued and appropriate therapeutic measures should be taken, if abnormalities are observed.

(2) Other adverse reactions

	Incidence unknown
**Hypersensitivity** Note 1)	Rash, Redness, Pruritus, Urticaria, etc.
**Gastrointestinal**	Anorexia, Epigastric distress, Nausea, Vomiting, Abdominal pain, Diarrhea, etc.

Note 1) If such symptoms are observed, administration should be discontinued.

5. Use in the Elderly

Because elderly patients often have reduced physiological function, careful supervision and measures such as reducing the dose are recommended.

6. Use during Pregnancy, Delivery or Lactation

The safety of TJ-108 in pregnant women has not been established. Therefore, TJ-108 should be used in pregnant women, women who may possibly be pregnant only if the expected therapeutic benefits outweigh the possible risks associated with treatment.

7. Pediatric Use

The safety of TJ-108 in children has not been established. [Insufficient clinical data]

8. Effects on Laboratory Tests

Treatment with TJ-108 may cause an increase in blood AG (1,5-anhydro-D-glusitol) level.

9. Other Precautions

Eczema, dermatitis, etc. may be aggravated.

PACKAGING

Bottles of 500 g
3.0 g × 42 packets
3.0 g × 189 packets

REQUEST FOR LITERATURE SHOULD BE MADE TO:

Consumer Information Services Center
Tsumura & Co.
2-17-11 Akasaka, Minato-ku, Tokyo 107-8521, Japan

Manufactured and Distributed by:

Tsumura & Co.
2-17-11 Akasaka, Minato-ku, Tokyo 107-8521, Japan
